# The Role of Semaphorins and Their Receptors in Gliomas

**DOI:** 10.1155/2012/902854

**Published:** 2012-09-23

**Authors:** Janice Wai Sze Law, Alan Yiu Wah Lee

**Affiliations:** Neurobiology/Ageing Programme, Department of Physiology, Yong Loo Lin School of Medicine, Life Sciences Institute, National University of Singapore, Centre for Life Sciences, 28 Medical Drive, Singapore 117456

## Abstract

Gliomas are the most common tumor in the central nervous system. High-grade glioblastomas are characterized by their high invasiveness and resistance to radiotherapy, leading to high recurrence rate and short median survival despite radical surgical resection. Characterizations of gliomas at molecular level have revealed aberrations of various growth factor receptors, receptor tyrosine kinases, and tumor suppressor genes that lead to deregulation of multiple signaling pathways, thereby contributing to abnormal proliferation, invasion, and resistance to apoptosis in cancer cells. Recently, accumulating evidence points to the emerging role of axon guidance molecules in glioma progression. Notably, many signaling events harnessed by guidance molecules to regulate cell migration and axon navigation during development are also found to be involved in the modulation of deregulated pathways in gliomas. This paper focused on the signalings triggered by the guidance molecule semaphorins and their receptors plexins and neuropilins, and how their crosstalk with oncogenic pathways in gliomas might modulate cancer progression. The emerging role of semaphorins and plexins as tumor suppressors or oncogenes is also discussed.

## 1. Introduction

Gliomas are the most common tumor in the central nervous system, with an incidence rate of 5 to 10 per 100,000 for the malignant form [1]. Based on their histological characteristics and expression of lineage markers, gliomas can be classified into astrocytoma, oligodendroglioma, and ependymoma [2]. Astrocytomas account for 60% of all primary brain tumors and the World Health Organization (WHO) classification system grades them on a scale of I to IV according to their increasing degree of malignancy [3]. Among them, Grade IV is one of the most highly invasive types of tumor [4], characterized by microvascular proliferation. Its aggressive infiltrative growth leads to an extremely high recurrence rate within a short period of time even after radical surgical resection. Worse still, many glioblastomas are found to be resistant to chemo- or radiotherapy due to DNA repair by the protein O6-methylguanine-methyltransferase (MGMT) and impaired apoptotic pathways. Median survival after initial diagnosis is therefore currently only around 12 to 18 months [5]. This apparently calls for a more thorough understanding of the pathoetiology at both cellular and molecular level to provide insight into the devise of novel and effective therapeutic treatments. This paper revisits the biological features of various genetic pathways deregulated in human gliomas, followed by an overview of the involvement of semaphorins and their receptors in these signalings that lead to their emerging role in the regulation of gliomagenesis.

## 2. Classification of Astrocytomas

Astrocytomas can be categorized into localized and diffuse forms. Localized astrocytic tumors exhibit a circumscribed growth pattern with limited capacity for parenchymal infiltration. Pilocytic astrocytomas for instance, are slow-growing tumors that occur primarily in children or young adults [6]. They are mostly nonaggressive and show little or no tendency to undergo anaplastic malignant transformation. This type of astrocytic tumor is classified as WHO grade I and is often curable if the tumor is resectable. Histologically, Rosenthal fibers are often observed on tumor sections. By contrast, diffuse astrocytomas are characterized by their high infiltration into peritumoral regions and dispersal to distant sites. Based on the WHO classification, they are subdivided into low-grade astrocytoma (grade II), anaplastic astrocytoma (grade III), and glioblastoma multiforme (GBM). 

Low-grade diffuse astrocytoma is characterized by slow growth and infiltration of neighboring brain structures [6]. Histologically, the tumors are characterized by low to moderate cellularity. Cancer cells are well-differentiated and show resemblance to astrocytes with little nuclear atypia. Also known as fibrillary astrocytomas, these cancers are believed to arise from neoplastic astrocytes. In fact, the cancer cells produce cytoplasmic processes that form a rich fibrillary stroma around the neoplasm, hence giving rise to a diffuse outline of the tumor in scans. These grade II tumors typically affect young adults (age of 25–50) and are treatable, with a mean survival of 6 years after surgical intervention. Nonetheless, the prognosis varies because of the tendency for these tumors to undergo malignant transformation to higher grades.

Anaplastic astrocytoma is highly proliferative and infiltrative. It is not uncommon that cells of these grade III malignant tumors invade along the white matter tracts and give rise to a classic “butterfly” pattern of spread at the corpus callosum. While histological characteristics of astrocytes can still be observed, the tumor cells become more pleomorphic with distinct nuclear atypia. They show increased mitotic activity and cellularity. Notably, anaplastic astrocytoma may develop *de novo* as primary tumor or arise from a grade II diffuse astrocytoma as secondary tumor. Afflicted patients are usually around the age of 40. Treatment regimens include surgical resection, radiation, and chemotherapy. Owing to the high invasiveness and aggressiveness, however, high recurrence rate is often observed within 2 years, during which the cancer typically would have progressed to high-grade glioblastoma.

GBM is the most invasive and malignant form of diffuse astrocytomas, and is one of the most aggressive type of human neoplasms. Histologically, the cancer cells are anaplastic and poorly differentiated. The pleomorphic astrocytic tumor cells show marked nuclear atypia and high mitotic activity. Rapid proliferation of the malignant cancer cells is accompanied by microvascular proliferation, though hypoxia is often resulted, leading to necrosis in the tumor core. In fact, neovascularization and necrotic cell death are the two major histopathological hallmarks for the diagnosis of glioblastomas versus anaplastic astrocytomas. Glioblastomas can be subdivided into primary or secondary form. Primary GBMs account for 60% of cases in adults above 45 years old. These tumors arise *de novo* with no sign of a preexisting less malignant precursor lesion. In contrast, secondary GBMs typically develop in patients below the age of 45 through malignant progression from lower grade diffuse astrocytoma or anaplastic astrocytoma in a span of one to ten years. At molecular level, the genetic lesions in primary and secondary GBMs also differ. Glioblastoma occurs most frequently in the cerebral hemisphere of adults between the ages of 45 and 70. Owing to the wide infiltration of glioblastoma cells, it is impossible to eradicate them by surgical means. In addition to their resistance to radio- and chemotherapy, recurrence rate of GBMs is close to 100%. A median survival of approximately one year is therefore commonly seen in the afflicted patients despite aggressive multimodality treatment regimens.

## 3. Genetic Pathways Deregulated in Glioblastomas

Although lower-grade gliomas (II and III) can undergo malignant progression to become secondary glioblastoma, most GBMs are diagnosed as *de novo* primary glioblastoma without sign of antecedent lower-grade tumor [7, 8]. Primary GBMs typically show gene amplification and mutations of the epidermal growth factor receptor (EGFR), deletion or loss-of-function mutations of the phosphatase and tensin homolog (PTEN) at chromosome 10, and less frequently gene amplification of the p53 inhibitor human double minute 2 (HDM2). Deregulation of these pathways is believed to contribute to the self-sustained proliferation, resistance to apoptosis, and highly invasive features of glioblastoma.

### 3.1. Hyperactivation of the EGFR-PI3K-AKT Pathway

**Figure 1:**
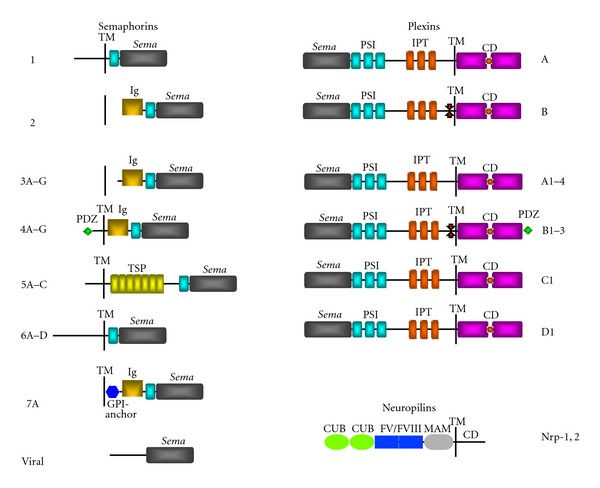
Semaphorins and their receptors. Schematic diagram to illustrate the structural features of different classes of semaphorins, plexins and neuropilins. Members of class 2 and 3 semaphorins are secreted, whereas semaphorins 1, 4–6 are single-pass transmembrane proteins. Class 7 semaphorin is membrane-bound via a GPI-anchor. All known semaphorins and plexins are characterized by the presence of a *sema* domain at the amino-terminus. While semaphorins are structurally diverse, plexin members are well-conserved and feature PSI and IPT domains in the extracellular moiety, and a split intracellular GTPase activating protein (GAP) domain. Plexin-Bs are distinctive by the presence of a PDZ domain binding site at the C-terminus. Neuropilins are transmembrane receptors that feature two complement binding (CUB) domains and FV/FVIII coagulating factor-like domain, and a MAM domain in the extracellular portion. Ig, immunoglobulin-like; TM, transmembrane domain; CD, cytoplasmic domain.

The EGFR gene is located on 7p12.1, which encodes a cell membrane receptor of the ErbB family. Upon activation by its ligands EGF or TGF*α*, EGFR undergo homodimerization to stimulate its intrinsic intracellular protein-tyrosine kinase activity. This triggers the downstream signaling cascades PI3K-AKT-GSK3*β*-Rac1 and the Ras-Raf-MEK-ERK (Figure 1) [9], which controls important functions such as cell survival, proliferation, and motility. Deregulation of EGFR, mostly in the form of gene amplification or overexpression is commonly found in primary GBMs [10]. The majority of GBMs with EGFR amplification also express the variant EGFRvIII [11], a mutant generated from an in-frame deletion of exons 2 to 7 that results in truncation of the extracellular domain. EGFRvIII is unresponsive to EGF stimulation but exhibits ligand-independent constitutive activity [12]. These genetic aberrations of EGFR cause activation of PI3K and AKT signalings, which promote glioma cell proliferation and tumorigenicity via p27 [13]. AKT can also phosphorylate and inhibit GSK3*β*, thereby diminishing proteasomal degradation of cyclin D1 and hence promoting cell cycle progression [14]. Notably, the levels of phospho-PI3K and phospho-AKT are inversely associated with that of cleaved caspase 3, suggesting the association of activated PI3K pathway with reduced level of apoptosis commonly observed in GBMs. This partly accounts for the resistance of glioblastomas to radiotherapy. Ironically, there is evidence that shows activation of EGFRvIII by radiation, posing a concern in prescribing radiotherapy as a treatment regimen in the management of glioblastomas [15]. Furthermore, PI3K and AKT/PKB can activate Rac1 [16], which in turn triggers TIMP2, MMP2, and MMP9 pathway to promote cell invasion [17]. 

### 3.2. Hyperactivation of the Ras-Raf-MEK-ERK Pathway Causes High Proliferation and Resistance to Apoptosis in Glioblastomas

In addition to PI3K signaling, activated EGFR also triggers the Ras-Raf-MEK-ERK pathway. In fact, several growth factor pathways that are aberrantly activated in glioblastomas, including platelet-derived growth factor (PDGF), insulin-like growth factor-1 (IGF-1), hepatocyte growth factor/scatter factor (HGF/SF), vascular endothelial growth factor (VEGF), and transforming growth factor-*β* (TGF-*β*) also converge to Ras activation. High level of activated Ras is always found in glioma cell lines and glioblastoma biopsies [18]. This is in contrast to many other cancer types where Ras is abnormally activated by oncogenic mutations. Active Ras recruits Raf kinase to the membrane, where it is primed by phosphorylation. Raf in turn activates MEK and ERK. Activated ERK then translocates into the nucleus to activate various transcription factors to induce cell cycle progression and antiapoptotic signalings [19]. Importantly, blockade of Ras activation in gliomas slows down cancer cell proliferation [18, 20]. Application of Ras inhibitor also reduces migration of U87-MG gliomas cells [21]. The deregulation of signalings in gliomas is further complicated by intensive cross-talk between the Ras and the PI3K pathways. Ras is known to stimulate PI3K through physical association, which activates PI3K-mediated antiapoptotic processes and hence contributes to Ras oncogenicity [22]. Conversely, PI3K was proposed to participate in ERK1/2 activation [23]. In summary, aberrant activation of specific receptor tyrosine kinases in gliomas elicits both the Ras and the PI3K cascades, leading to promotion of cancer cell proliferation, survival, motility, and resistance to apoptosis.

### 3.3. Loss of PTEN Functions Is Associated with Glioblastomas

Located at 10q23.3, the PTEN gene encodes a protein that contains a tensin like domain and a catalytic domain at the N-terminus similar to that of the dual-specificity protein tyrosine phosphatases [24, 25]. Interestingly, it was found that the phosphatase activity of PTEN exhibits substrate preference towards phosphoinositide such as phosphatidylinositol(3,4,5)-trisphosphate (PIP_3_) [26]. PIP_3_ is an important lipid product of PI3K that serves as second messenger for the activation of AKT/PKB, which promotes cell proliferation and survival through the inhibition of apoptotic cell death. The lipid phosphatase activity of PTEN in dephosphorylating PIP_3_ into PIP_2_ therefore serves as a negative regulator of PI3K signalings. In addition to cell proliferation and apoptosis, PTEN is also involved in the regulation of glioma cell motility. The central domain of PTEN shows protein tyrosine phosphatase activity, which directly dephosphorylates focal adhesion kinase (FAK) in integrin complex, hence inhibiting integrin-mediated migration and invasion [27–29]. Furthermore, PTEN was found to directly dephosphorylate the SH2-containing adaptor protein (Shc) upstream of Ras, hence downregulating the Ras/Raf/MEK/ERK pathway and inhibits cell spreading, migration, and invasion [28]. Taken together, PTEN modulates the integrin-FAK and the Shc-Ras-MAPK pathways, thereby regulating both random and directional cell motility in glioma cells, respectively [30]. 

Notably, a majority of primary glioblastoma and established glioma cell lines such as U87-MG and U373-MG carry mutations in the PTEN gene that result in diminished or null expression of the protein [25, 31, 32], leading to aberrant activation of AKT [33], FAK [34–36], and Ras [18, 37, 38] in GBMs. In fact, loss of heterozygosity (LOH) 10q is one of the most common genetic aberrations in GBMs. A complete loss of the entire chromosome 10, where the PTEN gene resides is typical for primary glioblastomas. The loss of PTEN check on PIP_3_ and AKT/PKB signaling pathway in glioma therefore spares the cells from apoptosis and promote cell cycle progression. Deregulation of PTEN functions could also render EGFR kinase-inhibitor therapy ineffective because of the dissociation of EGFR inhibition from downstream PI3K pathway inhibition. Conceivably, a compromise of PTEN functions in counteracting FAK and Ras signalings contributes to the high basal cell motility and the progression of glioblastoma [28, 30].

### 3.4. pRB Pathway

The retinoblastoma protein (pRB) is encoded by the RB1 gene located at 13q14.1. pRB is a tumor suppressor protein that blocks cell cycle progression through G1 into S-phase, hence preventing cells from replicating damaged DNA. Mechanistically, pRb forms complexes with transcription factors of the E2F family and suppresses the coordinated transcription of genes necessary for DNA synthesis [39]. The pRb-E2F complex also attracts histone deacetylase (HDAC) protein to the chromatin, reducing transcription of S phase promoting factors, further suppressing DNA synthesis. The cell cycle arrest function of pRB is counteracted upon phosphorylation by the cyclin-dependent kinases Cdk4 and Cdk6, which form complexes with cyclin D1, leading to a release of E2F and hence derepression of cell cycle progression. Under physiological conditions, the Cdk4/cyclin-D1 activity is checked by CDKN2A (p16^INK4a^) protein to prevent aberrant cell proliferation. In cancer states, however, an upregulation of cyclin D1 and Cdk4, or an inactivation of CDKN2A is often seen, leading to uncontrolled cell proliferation. In fact, the pRB pathway is commonly inactivated in both primary and secondary glioblastomas. Genetic loss or promoter methylation of RB1, homozygous deletion of CDKN2A, or amplification of CDK4 frequently occurs in GBMs [40, 41]. These deregulations contribute to uncontrolled cell cycle progression and tumorigenesis.

## 4. Involvement of Axon Guidance Molecules in Glioma Progression

In addition to deregulation of the classical growth factor receptors and signaling pathways, increasing evidence suggests the involvement of novel players in the progression of gliomas. Diffused gliomas are known to be highly infiltrative into the surrounding parenchyma of tumor mass even at low-grade. This poses an immense challenge to clinical management of the disease and high recurrence rate is commonly observed even after radical surgical resection of the tumor. Notably, clinical observations revealed a preferential invasion of gliomas along white matter tracts such as the corpus callosum [42]. Furthermore, cessation of glioma cell migration is often observed at the junction between the white and the grey matter. These findings suggest the presence of tropic and guidance cues for the invasion of gliomas within the CNS. In fact, accumulating evidence suggests the involvement of axon guidance molecules such as netrin [43], Slit [44], and semaphorins [45] in glioma progression. A recent study has demonstrated that netrin functions to inhibit glioma cell motility in an autocrine fashion [43]. A loss of expression of DCC (Deleted in Colorectal Carcinoma), the receptor for netrin has been found to correlate with glioma progression [46]. Similarly, Slit 2 inhibits glioma cell invasion in a Robo-dependent manner [44, 47], and its expression is epigenetically downregulated in gliomas [48]. Semaphorins are amongst the largest family of guidance molecules that trigger both repulsive and attractive axon guidance functions through different receptors and coreceptors. Growing body of evidence indicates that semaphorins also subserve pro-tumorigenic and tumor suppressor roles in various cancer types, including gliomas [49–52].

### 4.1. Semaphorins and Their Receptors Neuropilins and Plexins

Semaphorins, originally identified as guidance cues that navigate axons in the nervous system, fall into eight subclasses of secreted, membrane glycosylphosphatidyl-inositol (GPI)-anchored, and transmembrane proteins (Figure 1). Semaphorins are characterized by the presence of a *sema* domain and a PSI (plexins, semaphorins and integrins) domain at the N-terminus, which are also present in their receptors plexins [53]. Most family members harbor the immunoglobulin (Ig)-like domain, which is a signature feature found in the Ig superfamily (IgSF) of cell adhesion or recognition molecules. Class 5 semaphorins are distinctive due to the presence of multiple thrombospondin 1 (TSP1) domains, which has been implicated in platelet aggregation, angiogenesis, and tumorigenesis [54]. To date, class 3 semaphorins are the most thoroughly studied for their physiological and pathological functions. Interestingly, members of this class exist in secreted form and interact directly with the receptors neuropilin (Nrp)-1 or -2, which have small intracellular tail and need to engage plexins as coreceptors for signal transduction functions. 

Mammalian plexins are classified into four subfamilies plexin-A to -D [53], which share high homology in structural domains such as the *sema *domain and the cysteine-rich motifs related to c-Met proto-oncogene. Despite this, interactions between different subfamilies of plexins and semaphorins still show differential specificity, which trigger different sets of biological functions (Table 1). A second level of functional specificity is attained by the coupling of plexins with various coreceptors expressed in a cell- or tissue-specific manner, such as neuropilins, L1 adhesion molecule, off-track (Otk), c-Met proto-oncogene, the receptor tyrosine kinase ErbB2, CD72, and Tim-2 [55–59]. These lead to the implication of semaphorins and plexins in a vast array of biological processes beyond axon guidance, such as cell adhesion, cell motility, angiogenesis, immune responses, organogenesis, and tumorigenesis.

### 4.2. Semaphorins and Their Receptors in Tumorigenesis

The implication of semaphorins in cancer development was first suggested by the observation of a deletion of chromosomal region 3p21, where Sema3B and Sema3F reside, in almost all cases of small cell lung cancer (SCLC) [60–62]. Ensuing research revealed that many class 3 semaphorins exhibit tumor suppressor function by inhibiting proliferation and promoting apoptotic cell death in cancer cells. Sema3B, for instance induces apoptosis in lung and breast cancer [63, 64], whereas Sema3F shows antiproliferative and antiangiogenic functions in melanoma, lung, breast, and colorectal cancers [65, 66]. In addition, Sema3A and 3F were shown to inhibit cell migration of breast carcinoma and metastasis of melanoma, respectively, through their receptors neuropilins and plexins in an autocrine or paracrine manner [67, 68]. Sema3A/Nrp-1 and Sema3F/Nrp-2 interactions also block endothelial cell migration and initiate antiangiogenic signaling cascades [67]. While Sema3A, 3B, and 3F demonstrate antitumorigenic or antiangiogenic properties in various cancer types, other members are implicated in the promotion of tumor progression. Overexpression of Sema3C in prostate cancer cell lines promotes cell invasion [69]. An upregulation of Sema3E transcript has been observed in metastatic human lung adenocarcinoma cells [70]. Expression of Sema3E induces lung metastasis of mammary adenocarcinoma and promotes migration and growth of endothelial cells [71]. 

While the importance of semaphorins in cancer development is most well characterized in secreted class 3 members, transmembrane semaphorins of class 4, 5, and 6 begin to emerge as important players. Sema4D is highly expressed in cell lines derived from head and neck squamous cell carcinomas (HNSCCs). When shed from HNSCC cells, Sema4D stimulates endothelial cell migration. Silencing Sema4D expression by lentiviral shRNA dramatically reduces the size and vascularity of HNSCC tumor xenografts [72]. These findings suggest the angiogenic function of Sema4D. An overexpression of Sema4D was found to be significantly correlated with lymph node and distant metastasis of pancreatic ductal adenocarcinoma. *In vitro* experiments revealed that stimulation of pancreatic cancer cells with Sema4D triggers its receptor plexin-B1 and leads to the promotion of invasiveness through activation of the MAPK and the AKT pathway [73]. In prostate cancers, a significant upregulation of both Sema4D and plexin-B1 has been observed [74]. Mutations have also been found in plexin-B1 that causes an impairment of its R-RasGAP activity, hence increasing cell motility and invasion. In contrast to these oncogenic roles of Sema4D, however, various lines of evidence point to a tumor suppressor role of its receptor plexin-B1. For instance, a loss of plexin-B1 expression is associated with poor prognosis in ER+ breast cancer patients [75]. Plexin-B1 downregulation is also seen in renal cell carcinoma [76]. Tumor suppressor function of plexin-B1 has been demonstrated in melanoma cells, and its expression is downregulated by oncogenic B-Raf signaling via MEK/ERK pathway [77]. Divergent functions in cancer development is also implicated in class 6 semaphorins. Sema6A inhibits both bFGF/VEGF and tumor cell line-induced neovascularization [78], whereas an upregulation of Sema6D expression is associated with the development of gastric carcinoma [79]. A recent study has revealed the role of Sema7A in inhibiting cancer cell invasion by inactivation of cofilin through plexin-C1, which is consistent with the observation of a downregulation of Sema7A in metastatic melanoma cells [80]. Among other members, class 5 semaphorins are peculiar due to the presence of thrombospondin repeats in the extracellular domain, which have been implicated in cancer cell motility and proliferation [81]. The expression of semaphorin 5A (Sema5A) and its receptor plexin-B3 was shown to be associated with the aggressive nature of pancreatic, prostate, and gastric cancers [82–85]. Nonetheless, a recent report has demonstrated significant downregulation of Sema5A transcript and protein in nonsmall cell lung carcinoma, which correlates with poor survival of the patients, suggesting potential anticancer effect of Sema5A [86]. Consistent with this is a recent study that reports the presence a novel missense mutation in plexin-B3 in human pancreatic ductal adenocarcinomas, which prevents interaction with its ligand Sema5A, hence impairing their signaling and contributing to tumor progression [87].

### 4.3. Semaphorins and Their Receptors in Gliomagenesis

The implication of semaphorins, neuropilins, and plexins in gliomagenesis was suggested by their presence in human glioma cells [88]. In particular, the downregulation of Sema6B expression in two human glioblastoma cell lines when treated with retinoic acid, an antitumor and differentiation-inducing agent suggest its involvement in tumor formation [49]. To date, class 3 semaphorins remain the most well-characterized subfamily in the development of gliomas. An increase in expression of the Sema3B gene was found to be associated with poor prognosis and survival of glioma patients [50]. Yet a later study reported the opposite by demonstrating positive correlations of Sema3B, Sema3G and neuropilin-2 with prolonged survival in glioma patients [89]. Similarly, a protumorigenic role of Sema3A was implicated by its overexpression in human GBM specimens, which acts in an autocrine fashion to promote glioma cell dispersal through neuropilin-1. Silencing Sema3A expression by RNA interference resulted in a significant suppression in migration and an alteration in glioma cell morphology in a Rac1-dependent manner [90]. Nonetheless, it should be noted that Sema3A exhibits an antiangiogenic effect in meningiomas, as evident by the correlation of its high expression with reduced tumor vascularization. Recurrence of meniginomas is often associated with a decline in Sema3A expression [91]. In contrast to Sema3A, *in vitro* data revealed that Sema3F inhibits glioma cell migration via RhoA inactivation by p190 RhoGAP [92]. Frequent deletion of 3p21 in gliomas, where the Sema3F gene resides could abolish its effect in counteracting tumor cell motility. In a related study, low level of Sema3F expression was observed in highly metastatic human tumor cell lines due to Id2-mediated repression of gene transcription, which possibly accounts for the enhancement of tumor cell migration and invasion [93]. Similarly, an overexpression of or an exogenous stimulation by Sema3G in human U251 glioma cells is sufficient to inhibit migration and invasion, suggesting a cell autonomous or paracrine mechanism [94]. Recently, Sema5A has been shown to suppress human glioma cell migration and invasion in a plexin-B3- and Rac1-dependent manner [51]. A downregulation of Sema5A protein is found from low- to high-grade gliomas. Notably, stimulation of human glioblastoma cells with Sema5A promotes their differentiation from anaplastic to astrocytic morphology, which is reminiscent of the less pleomorphic and well-differentiated features associated with low-grade astrocytomas. Taken together, these findings suggest a tumor suppressor role of Sema5A, which is compromised towards high-grade glioblastomas [52]. In addition to semaphorins, there has been evidence pointing to the importance of neuropilins in glioma development. Analyses of human glioma specimens revealed a significant correlation of Nrp-1 expression with glioma progression to high grades. An upregulation of Nrp-1 in glioma cells was found to promote tumor growth and angiogenesis *in vivo*. These effects are mediated through the promotion of c-Met phosphorylation, hence potentiating the autocrine HGF/c-Met signaling pathway [95]. 

## 5. Semaphorin and Plexin Signalings

Semaphorins and their receptors are inarguably involved in the progression of a large number of cancer types, with functions ranging from cell migration, invasion, metastasis, proliferation, and apoptosis, to angiogenesis. It is not uncommon that many semaphorins can induce pro- and antitumorigenic effects in different cancers, making it a challenging task to decipher the underlying mechanisms of these activities. For instance, Sema3B induces apoptosis in lung and breast cancer [63, 64] but is associated with poor prognosis and survival of glioma patients [50]. Sema3A promotes glioblastoma cell dispersal but inhibits angiogenesis in meningiomas [90, 91]. Possible reasons for these diversities could be attributable to cross-interactions between different subfamilies of semaphorins and plexins, which show differential binding affinity and specificity, thereby triggering different sets of signaling events and functions. A second level of regulation is attained by coupling plexins and/or neuropilins to various coreceptors expressed in a cell- or tissue-specific manner, such as neuropilins, L1-cell adhesion molecule, c-Met proto-oncogene, the receptor tyrosine kinase ErbB2, and PDGFR. This could potentially elicit a more diverse network of signaling events.

### 5.1. Plexin-A Signaling Regulates PI3K Pathway

**Figure 2:**
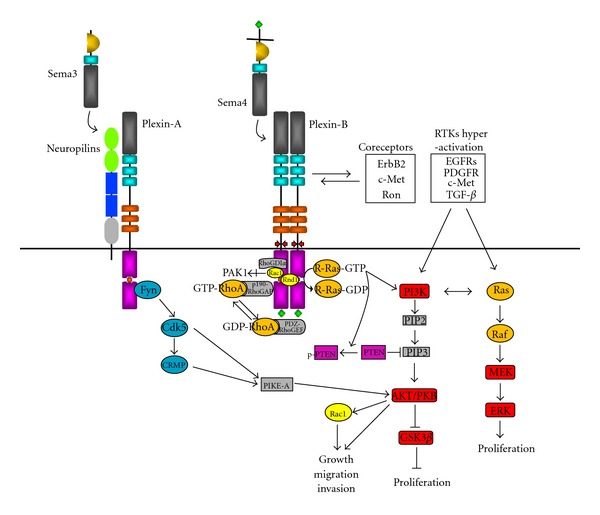
Signaling cascades mediated by plexin-A and plexin-B, and their crosstalk with oncogenic pathways in gliomas. Plexin-As serve as coreceptors for neuropilins to transduce signals of class 3 semaphorins. Fyn is activated upon semaphorin stimulation, which phosphorylates cyclin-dependent kinase-5 (Cdk5) and in turn collapse response mediator protein (CRMP). Cdk5 also phosphorylates isoform A of phosphatidylinositol 3-kinase enhancer (PIKE-A), which is a pro-oncogenic and antiapoptotic factor that activates AKT and promotes glioma cell growth, migration, and invasion. Stimulation of plexin-B with semaphorins causes recruitment of active Rac1 to the Cdc42/Rac interactive binding (CRIB) domain in the cytoplasmic tail, leading to sequestration of Rac1-GTP from its effector p21-activated kinase (PAK) and suppression of its downstream signalings. Stimulation of plexin-B also promotes the recruitment of Rac1 to Rho GDP-dissociation inhibitor (RhoGDI) *α*, which sequesters Rac1 and shuttles it from the membrane to the cytosol to prevent activation. The PDZ domain binding motif at the C-terminus of plexin-B interacts with PDZ domain containing Rho guanine nucleotide exchange factors (PDZ-RhoGEF), which promotes RhoA activation. RhoA-GTP can signal to Raf and activate the MAPK pathway. The GTPase activating protein (GAP) domain in the cytoplasmic tail of plexin-A and -B is activated upon semaphorin stimulation and Rnd1 binding, leading to inactivation of R-Ras and PI3K signaling but derepression of PTEN activities.

Sema3A signaling involves phosphorylation of plexin-As by nonreceptor tyrosine kinases Fes/Fps and Fyn. Fyn, a member of the Src family of tyrosine kinases, associates with and phosphorylates plexin-A2 in response to Sema3A (Figure 2). Active Fyn then phosphorylates cyclin-dependent kinase-5 (Cdk5) [96]. While activated Cdk5 is classically known to phosphorylate CRMP-2 (collapse response mediator protein) in neurons and leads to growth cone collapse, a recent report has shown that Cdk5-mediated phosphorylation of PIKE-A (Isoform A of phosphatidylinositol 3-kinase enhancer), a novel pro-oncogenic and antiapoptotic factor that activates AKT and promotes cancer cell growth, induces glioblastoma cell migration and invasion [97, 98]. These signalings are consistent with the protumorigenic role of Sema3A in promoting glioma cell dispersal [90]. Interestingly, a recent report has provided *in vivo* evidence that hyperactivated EGFR signaling in glioblastoma patients also recruits Fyn kinase to enhance tumor survival, growth and invasion [99].

### 5.2. Plexin-B Signaling Regulates RhoGTPases

Stimulation of plexin-B1 with Sema4D has been shown to recruit active Rac1 to the Cdc42/Rac interactive binding (CRIB) domain in the cytoplasmic tail [100]. This leads to sequestration of Rac1-GTP from its downstream effector p21-activated kinase (PAK), hence suppressing Rac1-mediated functions such as cell migration and proliferation. A recent report that investigates the significance of Sema5A and plexin-B3 in glioma has also shown their suppression of Rac1 activation, though through a different mechanism. Sema5A stimulation of plexin-B3 promotes the recruitment of Rac1 to Rho GDP-dissociation inhibitor (RhoGDI) *α*, which is a negative modulator that sequesters small GTPases and shuttles them from membrane to the cytosol to prevent activation (Figure 2). The inactivation of Rac1 was confirmed to contribute to Sema5A-induced impediment of glioma cell migration and invasion, as evident by the abolishment of effect upon forced expression of a constitutively active Rac1 mutant [51].

In addition to Rac1, plexin-Bs are also involved in the regulation of RhoA activation. Plexin-B members are peculiar to feature a PDZ domain binding motif at their carboxyl terminus. A number of studies have demonstrated direct interaction of plexin-B1 with two PDZ domain containing Rho guanine nucleotide exchange factors (RhoGEF), namely, PDZ-RhoGEF and leukemia-associated RhoGEF (LARG) [101–105]. Stimulation of plexin-B1 by Sema4D leads to RhoA activation in a PDZ-RhoGEF/LARG dependent manner, causing remodeling of actin cytoskeleton that is important for the regulation of cell morphology and cell motility [101, 102, 104]. Notably, Sema4D is known to activate the MAPK pathway, which is dependent on the C-terminus of plexin-B1 and LARG-mediated activation of RhoA [106]. In fact, RhoA-GTP was formerly shown to signal to Raf to activate MAPK cascade [107]. Taken together, this provides a link between the signalings mediated by Sema4D and MAPK. However, it should be emphasized that the activation of RhoA through PDZ-RhoGEF represents a specific mode restricted to members of the plexin-B subfamily due to the exclusive presence of PDZ domain binding motif in their C-terminus. On the other hand, there is also opposing evidence that indicates the role of Sema4D in RhoA inactivation instead. Stimulation of plexin-B1 with Sema4D was found to promote the binding of plexin-B1 with p190-RhoGAP, which inactivates RhoA and leads to disassembly of focal adhesions [108]. Interestingly, the GAP activity of p190-RhoGAP is enhanced by direct interaction with the constitutively active small GTPase Rnd1 [109], which also binds to the intracellular domain of plexin-B1 [110]. It remains to be determined whether plexin-B1 serves as a docking site to facilitate p190-RhoGAP activation by Rnd1 or instead functions as a negative regulator by sequestering Rnd1 from p190RhoGAP. 

### 5.3. Plexin Singaling Regulates Ras Activity

Sequence analysis of plexins revealed the presence of weakly conserved RasGAP motifs in the cytoplasmic domain. A closer examination revealed two subdomains C1 and C2 which harbor the primary arginine (Arg) finger and the secondary Arg/Lys motif, respectively, which are highly conserved in the catalytic domain of GAPs and are essential for promoting the GTP hydrolytic activity of target GTPases. The intervening region between C1 and C2 encompasses the binding site for active Rac1 and constitutively active Rnd1. In basal state, C1 and C2 domains in plexin-B1 undergo intramolecular interaction that prevents their association with Ras. Rnd1 binding to the linker region relieves the closed conformation and allows the plexin-B1-Rnd1 complex to interact with R-Ras. However, RasGAP activity is not triggered until Sema4D binds to Rnd-1-bound plexin-B1, which causes dimerization of the receptor. The inactivation of R-Ras by GAP activity of plexin-B1 contributes to the inhibition of neurite outgrowth and growth cone collapse in hippocampal neurons by Sema4D [111, 112]. Notably, this GAP domain is conserved throughout all plexin families and has been shown to mediate repulsion of axon outgrowth induced by Sema3A and plexin-A1 as well [113]. In contrast to other Ras family members that are commonly deregulated in gliomas, R-Ras only weakly activates Raf and the MEK/ERK pathway [114, 115]. Instead, R-Ras stimulates PI3K and AKT with an efficiency comparable to that of Ras (Figure 2) [114, 116]. R-Ras has been shown to promote breast carcinoma cell migration and invasion in a PI3K- and integrin-dependent manner [116]. Inactivation of R-Ras by the RasGAP activity of plexin-B1 triggered by Sema4D was shown to cause an inhibition of cell migration through suppression of PI3K and *β*1 integrin activity [117]. Recently, there has been evidence to show that the inactivation of R-Ras by Sema4D-induced RasGAP activity of plexin-B1 leads to PTEN dephosphorylation, thereby activating its inhibitory effect on PI3K signaling [118]. This provides a plausible mechanism by which plexin-Bs regulates the PI3K/AKT pathway. While the importance of R-Ras activity regulation by semaphorin and plexin in glioma progression is yet to be scrutinized, increasing R-Ras expression and activation is known to correlate with advanced grades of gliomas and invading glioblastoma cells [37]. In addition to R-Ras, Sema4D and plexin-B1 can also suppress M-Ras activity, which results in an inhibition of dendritic outgrowth and branching in primary cortical neurons [119]. Remarkably, this signaling was found to induce a suppression of ERK activity, though the mechanism remains known. It is notable that a recent study has reported Rap1 as a novel substrate for the GAP activity mediated by plexin-B1. Rap1 overexpression was observed in astrocytomas of all malignancy grades, and was 2 to 3 folds higher in grade IV GBMs than in lower grades or non-neoplastic brain [120]. The effect of GAP activity of plexin-B on Rap1 in the control of glioma progression, if any, warrants further investigations. 

## 6. Coupling of Plexin-B with c-Met and ErbB2 as Coreceptors

c-Met is a tyrosine kinase receptor that upon stimulation by hepatocyte growth factor/scatter factor (HGF/SF) regulates various cellular functions such as cell proliferation, migration, invasion, and morphogenesis through activation of various signalings such as the Ras and the PI3K/AKT pathways. They are commonly overexpressed in gliomas and contribute to enhanced tumorigenicity [121]. An inhibition of endogenous HGF/SF and c-Met in gliomas suppresses the malignant phenotype [122, 123]. To date, all known members of plexin-B subfamily have been shown to interact with c-Met and the related proto-oncogene Ron, even in the absence of their ligands [124, 125]. Binding of Sema4D to plexin-B1 stimulates the tyrosine kinase activity of c-Met and Ron, resulting in tyrosine phosphorylation of both receptors and promotion of invasive response in a liver progenitor cell line and a colon carcinoma cell line [124, 125]. By contrast, a recent study has reported the opposite in melanoma. Sema4D fails to activate c-Met in melanomas, not because of a significant downregulation of plexin-B1 in these cancer cells. Instead, reconstitution of plexin-B1 expression in melanomas inhibits HGF-dependent activation of c-Met and migration [126]. A plausible explanation for this observation could be the competition between plexin-B1 and HGF for c-Met binding. Importantly, restoring plexin-B1 expression in melanoma cells is sufficient to suppress tumor growth *in vivo*, suggesting a tumor suppressor role of plexin-B1 [77]. In fact, the contrasting effects of semaphorins and plexins in regulating HGF/c-Met signaling is not unexpected given the ability of plexins to modulate Ras and PI3K as described above (Figure 2).

In addition to c-Met, members of the plexin-B family were also found to stably associate with another receptor tyrosine kinase ErbB2. Binding of Sema4D to plexin-B1 stimulates the intrinsic tyrosine kinase activity of ErbB2, resulting in the phosphorylation of both receptors [57]. ErbB2-mediated phosphorylation of plexin-B1 is important for Sema4D-induced activation of RhoA through PDZ-RhoGEF [57]. Contrary to this, Sema4D induces an inactivation of RhoA in a c-Met dependent manner [127]. The reciprocal effect of c-Met and ErbB2 in plexin-B1-mediated regulation of RhoA activation status is believed to partially account for the pro- and antimigratory effects of Sema4D in breast carcinoma and other cancer cells, depending on the relative expression levels of these two receptors [127]. Notably, analysis of astrocytomas of different grades shows no gene amplification of ErbB2. The expression of ErbB2 protein is weaker than EGFR and is at barely detectable level by Western and immunocytochemistry analysis [128]. The relative importance of c-Met and ErbB2 in the regulation of glioma behaviors by semaphorin and plexin signaling remains to be determined.

## 7. Regulation of Semaphorins and Plexins by Oncogenic Pathways

While semaphorins and plexins impose their pro- and antitumorigenic functions in gliomas through the regulation of various signaling pathways, accumulating evidence suggests that they are also targets for modulation by some of the aberrant oncogenic pathways. The gene encoding for Sema3B for instance, was identified as a direct target of the tumor suppressor gene p53 that is commonly mutated in gliomas [129]. Ectopic expression of p53 in a p53-deficient glioblastoma cell line results in dramatic induction of Sema3B mRNA expression and a reduction in the number of colonies in anchorage-independent growth assays. These findings suggest that Sema3B mediates the tumor suppressor functions of p53, which is often diminished in gliomas [89].

Hyperactivation of Ras and its downstream signaling cascades is another commonly observed genetic lesion in different cancers including gliomas. Oncogenic mutations of B-Raf, one of the effectors of Ras is prevalent in melanomas and other cancers but is rarely detected in gliomas, which when present are mainly restricted to high-grade malignant glioblastomas [130]. In gliomas, gain in copy number is more common than mutations for Ras and Raf [131], though both forms of deregulation lead to activation of the MEK/ERK pathway [132, 133]. Interestingly, constitutive B-Raf/MEK/ERK signaling was found to be associated with a downregulation of plexin-B1 transcript in melanoma [77]. Restoring plexin-B1 expression in melanoma cells is sufficient to suppress anchorage-independent growth *in vitro* and inhibit tumor growth in xenograft transplants in nude mice. The antitumorigenic effect of plexin-B1 is dependent on its R-RasGAP activity and is associated with AKT inactivation [77]. While R-Ras is less oncogenic than other Ras, its deregulation in gliomas is known to promote migration and invasion behaviors [37]. It is plausible that the antitumorigenic functions of plexin-B1 observed in melanoma are adopted in gliomas as well.

## 8. Concluding Remarks

Gliomas are known to exhibit not only histological heterogeneity but also variations in genetic alterations both inter- and intratumorally [134]. These have led to the proposition to incorporate molecular signature to classical histopathological diagnosis so as to provide a more informative description of the features of different glioma types. Here, an overview of various signaling pathways commonly deregulated in gliomas was presented. Despite our increasing knowledge of how these oncogenic pathways interact in a complex manner to contribute to aberrant cell proliferation, resistance to apoptotic cell death, enhanced migration and invasion, and induction of angiogenesis in glioma cells, the options of clinical treatment are still currently largely limited to a combination of surgical resection, followed by radiotherapy or chemotherapy using nonspecific alkylating agents such as temozolomide. While a number of inhibitors and antibodies that suppress the activities of deregulated kinases such as EGFR, c-MET, PDGFR, and PI3K are currently in difference phases of preclinical characterizations or clinical trials, the outcomes show great variations and are complicated by toxicity associated with the agents. The emerging roles of semaphorins and their receptors in the regulation of gliomagenesis and progression therefore represent potential novel candidate targets. Given the engagement of plexins with some of the oncogenic receptor tyrosine kinases such as c-Met and ErbB2 as coreceptors, a better understanding of the crosstalk between these signaling networks is required. Supported by the recent success in detecting MGMT methylation level in glioblastoma patients to predict their sensitivity to chemoradiotherapy using alkylating agents, future direction in clinical management of gliomas, which are heterogeneous in nature, is to define the molecular and genetic profile of the tumor in individual patient so as to devise a customized regimen of treatments.

## Figures and Tables

**Table 1:** List of semaphorins and their receptors.

Semaphorins	Plexins/Neuropilins	Coreceptors
Class 1		
Sema1a	PlexinA	Otk
Sema1b	PlexinA	Otk
Class 2		
Sema2a	PlexinB	—
Class 3		
Sema3A	Nrp-1; Plexin-A4	L1-CAM
Sema3B	Nrp-1, -2	Nr-CAM
Sema3C	Nrp-1, -2; Plexin-A2, -D1	—
Sema3D	Nrp-1	—
Sema3E	Plexin-D1	—
Sema3F	Nrp-1, -2; Plexin-A1, -A2	Nr-CAM
Sema3G	Nrp-2	—
Class 4		
Sema4A	Plexin-B1, -B2, -B3, -D1	Tim-2
Sema4B	Plexin-B1	—
Sema4C	Plexin-B2	—
Sema4D	Plexin-B1, -B2	c-Met; ErbB2; CD72
Sema4E	—	—
Sema4F	—	—
Sema4G	Plexin-B2	—
Class 5		
Sema5A	Plexin-B3, -A1, -A3	c-Met; ErbB2; CSPG; HSPG
Sema5B	Plexin-A1, -A3	—
Sema5C	—	—
Class 6		
Sema6A	Plexin-A4	—
Sema6B	Plexin-A4	—
Sema6C	Plexin-A1	—
Sema6D	Plexin-A1	VEGFR; NrCAM; Otk
Class 7		
Sema7A	Plexin-C1	*β*1-integrin
Class V		
Poxvirus A39R	Plexin-C1	—
